# Spinal Leptomeningeal Dissemination of Pineoblastoma in a 16-Year-Old Girl: A Case Report and Review of the Literature

**DOI:** 10.7759/cureus.110625

**Published:** 2026-06-10

**Authors:** Boutaina Agdi, Rania Chakir, Sara Harbaj, Karima Nouni, Lachgar Amine, Hanane Elkacemi, Tayeb Kebdani, Khalid Hassouni

**Affiliations:** 1 Radiation Oncology, National Institute of Oncology, Faculty of Medicine and Pharmacy - University Mohammed V, Rabat, MAR; 2 Radiotherapy, National Institute of Oncology, Faculty of Medicine and Pharmacy - University Mohammed V, Rabat, MAR

**Keywords:** case report, craniospinal radio-chemotherapy, medullary metastatic, penialoblastoma, rare tumor

## Abstract

Pineoblastoma is a rare and highly aggressive embryonal tumor of the pineal region, predominantly affecting children and adolescents. It is classified as a CNS WHO grade 4 tumor and is characterized by a high risk of cerebrospinal fluid dissemination. Although leptomeningeal spread is a recognized feature of this tumor, spinal metastatic involvement at diagnosis remains uncommon and indicates high-risk disease.

We report the case of a 16-year-old girl with no relevant past medical history who presented with a two-week history of sleep disturbances, right-sided strabismus, and projectile vomiting. Brain MRI demonstrated a heterogeneous pineal region mass measuring approximately 28 × 26 mm, associated with triventricular hydrocephalus. The patient underwent endoscopic ventriculocisternostomy and stereotactic biopsy. Histopathological and immunohistochemical findings confirmed the diagnosis of pineoblastoma. Staging spinal MRI revealed multiple dorsal spinal leptomeningeal nodules, consistent with CSF dissemination and spinal drop metastases.

Given the rarity of pineoblastoma and the absence of a universally established pineoblastoma-specific therapeutic protocol, the case was discussed with an international pediatric neuro-oncology expert. Based on this expert recommendation, the patient was treated using a high-risk medulloblastoma-derived regimen. She received craniospinal irradiation with weekly vincristine, followed by adjuvant chemotherapy with cisplatin, cyclophosphamide, and vincristine according to an ACNS0332-based approach. At three months after completion of treatment, follow-up MRI showed complete regression of the spinal metastatic lesions and a small residual cystic lesion in the pineal region. Clinically, the patient showed marked improvement, particularly in oculomotor function.

This case highlights the importance of systematic craniospinal staging at diagnosis in pineoblastoma and illustrates the potential value of early multidisciplinary and multimodal management in metastatic disease. At nine months of follow-up, the patient remained clinically stable, without evidence of spinal disease progression on serial imaging. However, this follow-up remains limited, and long-term clinical and radiological surveillance is essential because of the high risk of recurrence.

## Introduction

Pineoblastoma is a rare and highly malignant embryonal tumor arising from the pineal gland, a small endocrine structure located in the deep midline of the brain. It belongs to the spectrum of pineal parenchymal tumors and is classified as a WHO grade 4 CNS tumor because of its aggressive biological behavior, high proliferative potential, and tendency for CSF dissemination [1-3]. Pineal region tumors represent a small proportion of pediatric brain tumors, and pineoblastoma remains one of the rarest and most aggressive entities within this anatomical location.

The clinical presentation is usually related to mass effect and obstructive hydrocephalus caused by compression of the aqueduct of Sylvius. Patients may present with headache, nausea, vomiting, visual disturbances, Parinaud syndrome, oculomotor abnormalities, gait disturbance, or other signs of raised intracranial pressure [4]. Because the pineal region is adjacent to the third ventricle and CSF pathways, tumor cells may disseminate through the neuraxis, leading to leptomeningeal or spinal drop metastases. This biological behavior makes complete neuraxial staging essential at diagnosis, including brain and spinal MRI and CSF cytology when clinically feasible [5].

The differential diagnosis of pineal region masses is broad and includes germ cell tumors, pineocytoma, pineal parenchymal tumor of intermediate differentiation, glioma, lymphoma, atypical teratoid/rhabdoid tumor, and metastatic disease. Therefore, histopathological and immunohistochemical confirmation is required before definitive treatment. Contemporary molecular studies have also shown that pineoblastoma is heterogeneous, with molecular subgroups involving alterations in microRNA-processing genes such as DICER1, DROSHA, and DGCR8, supporting the relevance of molecular profiling when available [6, 7].

Because pineoblastoma is extremely rare, there is no universally accepted standard therapeutic protocol specifically dedicated to metastatic disease. Management is usually individualized and may include treatment of hydrocephalus, maximal safe resection or biopsy, craniospinal irradiation, and systemic chemotherapy. In practice, treatment strategies are often extrapolated from protocols used for other high-risk pediatric embryonal CNS tumors, particularly medulloblastoma, because of shared aggressive behavior and a tendency for CSF dissemination [6, 7].

We report the case of a 16-year-old girl with pineoblastoma presenting with spinal leptomeningeal dissemination at diagnosis. This case emphasizes the importance of complete neuraxial staging, careful radiological characterization of spinal lesions, multidisciplinary decision-making, and cautious interpretation of early radiological response.

## Case presentation

A 16-year-old girl with no relevant past medical or family history presented with a two-week history of progressive sleep disturbances, right-sided strabismus, and recurrent projectile vomiting. There was no reported headache, seizure, fever, or altered level of consciousness. Neurological examination revealed oculomotor abnormalities, predominantly involving the right eye, without focal motor weakness, sensory deficit, or cerebellar signs.

A brain MRI confirmed a well-circumscribed, rounded pineal region mass measuring 23 × 21 × 25 mm. The lesion showed slight T1 hypointensity, T2 isointensity relative to the cerebral cortex, peripheral calcification on susceptibility-weighted imaging, and homogeneous gadolinium enhancement. It compressed the aqueduct of Sylvius and was responsible for active triventricular hydrocephalus with periventricular T2-weighted/fluid-attenuated inversion recovery (T2/FLAIR) hyperintensity consistent with transependymal CSF resorption. Perfusion sequences showed no intratumoral hyperperfusion, and no diffusion abnormality was identified (Figure 1).

**Figure 1:**
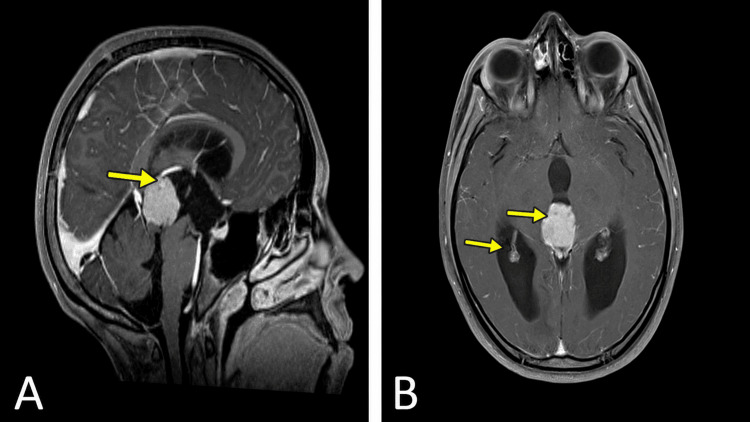
Sagittal (A) and axial (B) T2-weighted MRI showing a 28 × 26 mm pineal region mass (arrow) with triventricular hydrocephalus.

Given the presence of symptomatic obstructive hydrocephalus, the patient underwent endoscopic third ventriculostomy (ETV) for CSF diversion, combined with stereotactic biopsy of the pineal lesion for tissue diagnosis. The postoperative course was uneventful, with improvement in vomiting and symptoms related to intracranial hypertension. CSF cytology was not performed, which represents a limitation for complete metastatic staging.

As part of the staging workup, spinal MRI revealed multiple dorsal spinal leptomeningeal nodules, consistent with CSF dissemination and spinal drop metastases (Figure 2).

**Figure 2:**
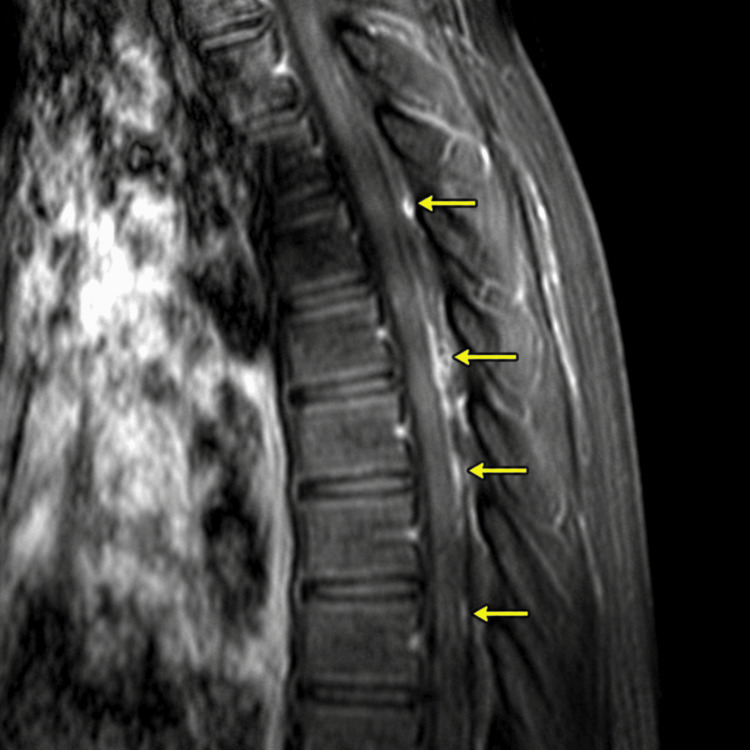
Sagittal T2-weighted MRI of the spine showing multiple dorsal spinal leptomeningeal lesions (arrows), consistent with metastatic involvement.

CSF cytology was not available, which limits complete metastatic staging. However, the presence of spinal leptomeningeal nodules on MRI supported metastatic disease at diagnosis.

Histological examination showed a proliferation of small basophilic round cells arranged in sheets, with rosette-like structures around fibrillary material. Tumor cells had round to elongated hyperchromatic nuclei, with mitotic figures, apoptotic bodies, and focal necrosis. Immunohistochemistry showed strong synaptophysin positivity, focal glial fibrillary acidic protein positivity, focal neurofilament positivity, retained INI1 expression, and an elevated Ki-67 proliferation index estimated at approximately 30%. Retained INI1 expression helped argue against atypical teratoid/rhabdoid tumor. Chromogranin, CRX, and OTX2 status were not available. Overall, the morphological and immunohistochemical findings were consistent with pineoblastoma.

The case was discussed in a multidisciplinary neuro-oncology setting. Given the rarity of pineoblastoma, the metastatic presentation, and the absence of a universally established pineoblastoma-specific therapeutic protocol, expert advice was sought from an international pediatric neuro-oncology specialist. Based on this expert recommendation, the patient was treated using a high-risk medulloblastoma-derived regimen, as pineoblastoma and medulloblastoma are both aggressive central nervous system tumors with a propensity for CSF dissemination.

The patient received craniospinal irradiation using volumetric modulated arc therapy (VMAT), combined with weekly vincristine, followed by adjuvant chemotherapy with cisplatin, cyclophosphamide, and vincristine according to an ACNS0332-based high-risk medulloblastoma-derived approach (Table 1). The radiotherapy treatment plan demonstrated coverage of the craniospinal axis, including the cranial compartment, spinal canal, and pineal tumor region (Figure 3).

**Table 1:** Summary of the ACNS0332-based treatment approach used for the patient. The patient was treated using an ACNS0332-based high-risk medulloblastoma-derived approach, combining craniospinal radiotherapy with weekly vincristine, followed by adjuvant chemotherapy. VMAT: Volumetric modulated arc therapy; Gy: Gray; N/A: Not applicable.

Phase	Treatment	Dose/schedule	Purpose/comments
Initial neurosurgical management	Endoscopic third ventriculostomy and stereotactic biopsy	N/A	Cerebrospinal fluid diversion and histological confirmation
Craniospinal radiotherapy	Craniospinal radiotherapy using VMAT	36 Gy in 20 fractions	Treatment of the craniospinal axis, including spinal metastatic lesions
Local boost	Boost to the pineal region using VMAT	To a total dose of 55.8 Gy in 31 fractions	Dose escalation to the primary tumor site
Concomitant chemotherapy	Vincristine	1.5 mg/m² weekly	Administered during radiotherapy
Adjuvant chemotherapy	Cisplatin, cyclophosphamide, and vincristine	Six cycles according to an ACNS0332-based high-risk medulloblastoma-derived regimen	Post-radiotherapy systemic chemotherapy

**Figure 3:**
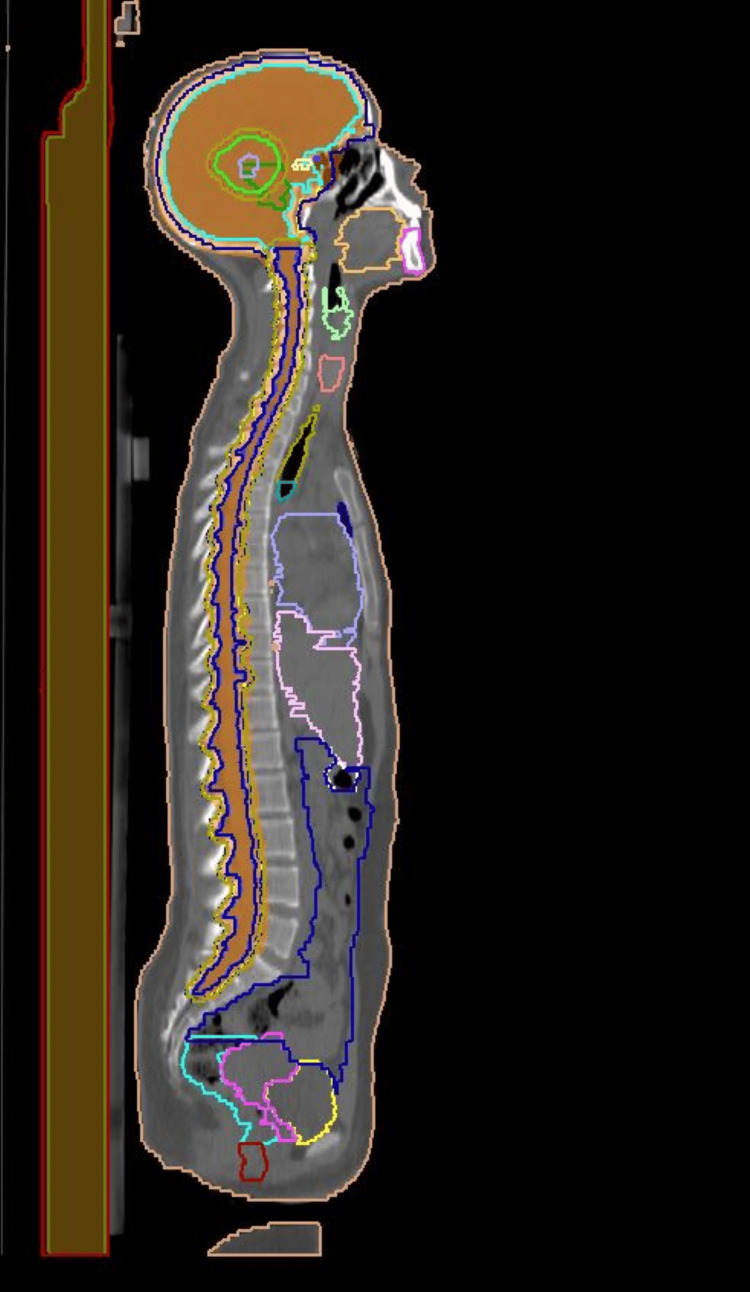
VMAT craniospinal irradiation treatment plan. Sagittal dosimetric image showing dose coverage of the craniospinal axis, including the brain, spinal canal, and pineal tumor region. VMAT: Volumetric modulated arc therapy.

At three months after completion of treatment, follow-up MRI demonstrated complete radiological regression of the previously described spinal leptomeningeal nodules and a small residual cystic lesion in the pineal region (Figure 4). Because enhancement characteristics were not fully available, this lesion was not interpreted as a definite residual tumor, and close radiological surveillance was planned (Figure 5).

**Figure 4:**
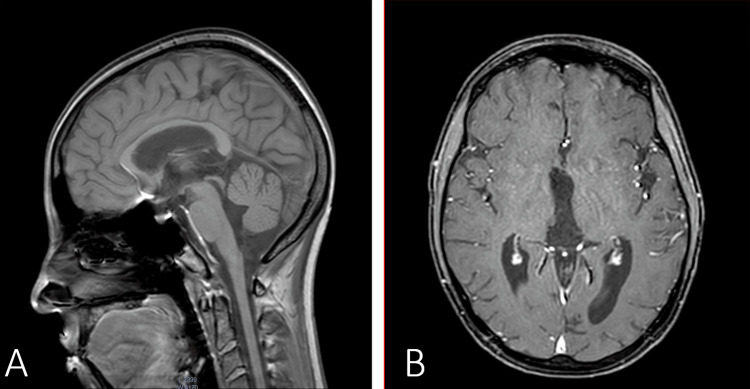
Follow-up sagittal (A) and axial (B) brain MRI images obtained three months after treatment showing a small residual cystic lesion in the pineal region.

**Figure 5:**
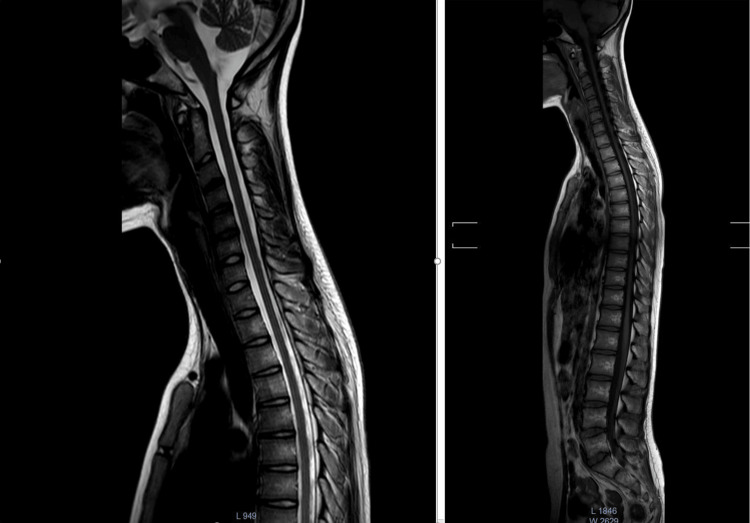
Follow-up sagittal spinal MRI performed after craniospinal radiochemotherapy showing complete radiological regression of the previously identified dorsal spinal leptomeningeal nodules.

Clinically, the patient showed marked improvement, particularly in oculomotor function, and no new neurological deficits were observed. She remains under regular neuro-oncological surveillance with serial brain and spinal imaging.

## Discussion

Pineoblastoma is one of the most aggressive tumors of the pineal region and belongs to the spectrum of pineal parenchymal tumors. Its rarity, particularly in children and adolescents, makes diagnosis and management challenging. Clinical manifestations are often nonspecific and are usually related to obstructive hydrocephalus or compression of adjacent midbrain structures. In the present case, projectile vomiting and oculomotor abnormalities were mainly consistent with raised intracranial pressure and pineal region mass effect rather than symptoms directly related to spinal leptomeningeal dissemination.

MRI plays a central role in the diagnostic workup of pineal region tumors. Pineoblastomas usually appear as heterogeneous pineal region masses and may be associated with obstructive hydrocephalus. However, imaging alone is not sufficient for definitive diagnosis because the differential diagnosis of pineal region masses is broad and includes germ cell tumors, pineocytoma, pineal parenchymal tumor of intermediate differentiation, glioma, lymphoma, atypical teratoid/rhabdoid tumor, and metastatic disease [4]. Therefore, histopathological and immunohistochemical confirmation remains essential before initiating definitive oncological treatment.

In this patient, the diagnosis was confirmed by stereotactic biopsy. Histological examination showed a densely cellular malignant tumor composed of small undifferentiated cells with hyperchromatic nuclei, scant cytoplasm, rosette-like structures, mitotic figures, apoptotic bodies, and focal necrosis. Immunohistochemistry showed strong synaptophysin positivity, focal glial fibrillary acidic protein positivity, focal neurofilament positivity, retained INI1 expression, and an elevated Ki-67 proliferation index estimated at approximately 30%. The Ki-67 index supports increased proliferative activity, although it should not be considered exceptionally high. Retained INI1 expression is diagnostically relevant because it argues against atypical teratoid/rhabdoid tumor, an important differential diagnosis among pediatric embryonal central nervous system tumors. Chromogranin, CRX, and OTX2 status were not available in this case, which represents a diagnostic limitation.

A key characteristic of pineoblastoma is its tendency to disseminate through CSF pathways. For this reason, complete neuraxial staging is required at diagnosis, including brain and spinal MRI and CSF cytology when clinically feasible [5]. In our patient, spinal MRI revealed multiple dorsal spinal leptomeningeal nodules, consistent with CSF dissemination and spinal drop metastases. This terminology is more precise than the broad term “spinal metastases,” as it distinguishes leptomeningeal nodular dissemination from intramedullary, epidural, or osseous metastatic lesions. The identification of leptomeningeal nodules at diagnosis directly influenced risk stratification and supported the indication for craniospinal irradiation. Metastatic presentation has been associated with poorer outcomes in pineoblastoma, emphasizing the prognostic importance of complete staging [1, 2].

The presence of spinal leptomeningeal dissemination indicates high-risk disease and is associated with poorer prognosis. Nandoliya KR et al. reported that metastatic presentation, less-than-gross total resection, chemotherapy without radiation, and age younger than three years were associated with poorer survival in pineoblastoma [1]. Hansford JR et al., in a pooled cohort of 178 pediatric pineoblastoma cases, also reported substantially worse outcomes in younger children and highlighted the need for collaborative studies because of the rarity of the disease [2]. Park T et al. emphasized the prognostic relevance of tumor grade and malignant behavior in pineal parenchymal tumors [3]. Extraneural or osseous dissemination is exceedingly rare; Constantine C et al. described osseous metastasis of pineoblastoma and emphasized the aggressive behavior of metastatic disease [8]. Other clinical series have also emphasized the prognostic relevance of tumor dissemination, age at diagnosis, extent of surgical resection, and use of adjuvant radiotherapy and chemotherapy [9-11].

The management of metastatic pineoblastoma requires an individualized multimodal approach. Neurosurgical intervention is often necessary to treat hydrocephalus and obtain tissue diagnosis. In the present case, ETV allowed CSF diversion, while stereotactic biopsy provided histological confirmation. Gross total resection may improve local control when safely feasible, but complete resection is not always possible because of the deep location of the pineal gland and its proximity to critical venous and midbrain structures.

Craniospinal irradiation is a key component of treatment in metastatic pineoblastoma because of the risk of microscopic and macroscopic CSF dissemination [5, 9]. In the present case, craniospinal irradiation was delivered using VMAT, allowing coverage of the craniospinal axis while optimizing dose distribution. The use of craniospinal irradiation was justified by the presence of dorsal spinal leptomeningeal nodules at initial staging. This approach is consistent with treatment strategies reported in high-risk pineoblastoma and other high-risk embryonal central nervous system tumors, where craniospinal irradiation combined with chemotherapy is commonly used [6, 7, 9-11].

Because pineoblastoma is extremely rare, no universally accepted pineoblastoma-specific therapeutic protocol exists, particularly for metastatic disease. Therefore, therapeutic approaches are commonly adapted from protocols used in other high-risk pediatric embryonal central nervous system tumors, particularly medulloblastoma [6, 7]. This approach is clinically reasonable because both pineoblastoma and medulloblastoma are aggressive embryonal tumors with a marked tendency for CSF dissemination. In the present case, the therapeutic strategy was selected after consultation with an international pediatric neuro-oncology expert. The use of an ACNS0332-based high-risk medulloblastoma-derived approach was considered appropriate because it combines craniospinal irradiation with weekly vincristine and adjuvant chemotherapy, thereby targeting both the primary tumor and neuraxial dissemination [6]. However, this should be interpreted as an expert-guided extrapolation from high-risk embryonal tumor management rather than as a pineoblastoma-specific standard protocol. In addition, ACNS0332-related data included patients with histologically diagnosed CNS-PNET and pineoblastoma, supporting its relevance as a broader high-risk embryonal central nervous system tumor framework while also highlighting the molecular and clinical heterogeneity of these tumors [7].

The radiological response observed at three months, with complete regression of the previously described spinal leptomeningeal nodules, was encouraging. At the latest nine-month follow-up, the patient remained clinically stable, without evidence of spinal disease progression on serial imaging. However, this follow-up period remains limited for such an aggressive tumor. Therefore, the favorable response should be interpreted cautiously as an early treatment response rather than evidence of durable disease control. Long-term clinical and radiological surveillance remains mandatory because pineoblastoma is associated with a high risk of recurrence, particularly in metastatic cases [1, 2, 9-11].

A comparative summary of selected studies included in the literature review is presented in Table 2. These studies highlight the aggressive nature of pineoblastoma, the prognostic impact of metastatic dissemination, and the need for multimodal therapy.

**Table 2:** Comparative analysis of selected studies included in the literature review. CNS-PNET: CNS primitive neuroectodermal tumor.

Study	Type of study	Population / cases	Focus / metastatic pattern	Treatment / management	Main findings
Nandoliya KR et al. [1]	Systematic review and individual patient data analysis	Published pinealoblastoma cases	Prognostic factors and survival trends	Surgery, radiotherapy, chemotherapy, or combined multimodal treatment depending on reported cases	Identified metastatic presentation, less-than-gross total resection, chemotherapy without radiation, and young age as poor prognostic factors
Hansford JR et al. [2]	Pooled outcome study	178 pediatric pinealoblastoma cases	Pediatric outcomes and age-related prognosis	Multimodal treatment, including surgery, radiotherapy, and chemotherapy according to institutional or cooperative group protocols	Reported poorer outcomes in younger children and emphasized the rarity of pinealoblastoma and the need for collaborative studies
Park TH et al. [3]	Retrospective single-institution study	Pineal parenchymal tumors	Survival and malignant transformation	Surgery with adjuvant radiotherapy and/or chemotherapy depending on tumor grade and clinical setting	Reported poorer outcomes in high-grade pineal parenchymal tumors and emphasized the prognostic relevance of tumor grade
Gaillard F and Jones J [4]	Review article	Pineal region masses	Clinical and imaging features	Diagnostic imaging workup; treatment depends on tumor type and histological confirmation	Described clinical presentation and radiographic features of pineal region tumors
Cocito C et al. [5]	Review article	Pediatric brain tumors	Leptomeningeal dissemination	Staging and treatment strategies adapted to tumor type, including craniospinal approaches when indicated	Supported the importance of recognizing and staging leptomeningeal dissemination in pediatric brain tumors
Leary SE et al. [6]	Randomized clinical trial	Children with high-risk medulloblastoma	High-risk medulloblastoma treatment framework	Craniospinal irradiation with chemotherapy, including vincristine during radiotherapy and adjuvant chemotherapy	Provides support for the ACNS0332-based treatment backbone, although not specific to pinealoblastoma
Hwang EI et al. [7]	Children’s Oncology Group ACNS0332 report	Patients with histologically diagnosed CNS-PNET and pineoblastoma	Molecular and clinical heterogeneity in ACNS0332	Treatment within the ACNS0332 high-risk embryonal CNS tumor framework	Supports the relevance of ACNS0332 as a broader high-risk embryonal CNS tumor framework
Constantine C et al. [8]	Case report and literature review	One case of pineoblastoma with osseous metastasis	Osseous / extraneural metastasis	Multimodal management details vary according to metastatic burden and clinical condition	Highlighted the rarity of osseous metastasis and the aggressive behavior of metastatic disease
Biswas A et al. [9]	Retrospective clinical experience and literature review	Pinealoblastoma patients	Treatment outcomes and patterns of failure	Surgery, craniospinal radiotherapy, and chemotherapy	Emphasized the aggressive nature of pinealoblastoma and the need for multimodal treatment
Tian Y et al. [10]	Retrospective study	18 pediatric pinealoblastoma cases	Clinical characteristics and prognostic factors	Surgery followed by radiotherapy and/or chemotherapy according to age, extent of disease, and clinical status	Reported clinical characteristics, therapeutic aspects, and prognostic factors in childhood pinealoblastoma
Kang YM et al. [11]	Single-institution experience	Pediatric pinealoblastoma cases	Pediatric treatment outcomes	Multimodal treatment including surgery, radiotherapy, and chemotherapy	Reinforced the role of aggressive multimodal management in pediatric pinealoblastoma

A comparative summary of selected studies included in the literature review is presented in Table 2. These studies highlight the rarity and aggressive behavior of pineoblastoma, the adverse prognostic impact of dissemination, the frequent use of multimodal treatment strategies, and the limitations of current evidence, which remains largely based on retrospective series, pooled analyses, and case reports.

This case has several limitations. First, the follow-up period remains relatively short, even with the updated nine-month follow-up, and does not allow definitive conclusions regarding long-term disease control. Second, CSF cytology was not available, which limits complete metastatic staging, as CSF assessment remains an important component of staging in embryonal central nervous system tumors with leptomeningeal dissemination [5]. Third, molecular profiling, including DICER1, DROSHA, DGCR8, and MYC status, was not available, although molecular heterogeneity is increasingly recognized as relevant in pineoblastoma classification and prognosis [7]. Fourth, immunohistochemistry for chromogranin, CRX, and OTX2 was not available. Finally, detailed chemotherapy toxicity data, including hematological, renal, audiological, and neurological tolerance, were not fully documented. These limitations reflect the challenges of reporting and managing rare tumors, for which evidence remains limited and often based on retrospective series, pooled analyses, and case reports [1, 2, 9-11].

This case also underscores the value of multidisciplinary and international collaboration in the management of rare pediatric brain tumors. Discussion within a neuro-oncology team and consultation with experienced pediatric specialists can help guide individualized treatment strategies, particularly when unusual metastatic patterns are identified.

## Conclusions

Pineoblastoma is a rare and aggressive pediatric CNS tumor with a marked tendency for CSF dissemination. This case highlights the importance of complete neuraxial staging at diagnosis, including brain and spinal MRI and CSF cytology when feasible, particularly when leptomeningeal dissemination is suspected.

Because no universally accepted pineoblastoma-specific therapeutic protocol exists, especially in metastatic cases, management should be individualized within a multidisciplinary framework. High-risk medulloblastoma-derived regimens may be considered after expert discussion, but they should be interpreted as extrapolated approaches rather than established pineoblastoma-specific standards.

The favorable radiological response observed in this patient, together with clinical stability at nine months of follow-up, is encouraging. However, this follow-up period remains limited for such an aggressive tumor and cannot be considered evidence of durable disease control. Long-term clinical and radiological surveillance, toxicity monitoring, and molecular characterization are essential to better define prognosis and guide future treatment strategies.
